# An Integrated Multi-Source Dataset for Measuring Settlement Evolution in the United States from 1810 to 2020

**DOI:** 10.1038/s41597-024-03081-x

**Published:** 2024-03-07

**Authors:** Yoonjung Ahn, Stefan Leyk, Johannes H. Uhl, Caitlin M. McShane

**Affiliations:** 1 University of Kansas, Department of Geography & Atmospheric Science, Lawrence, KS USA; 2 Institute of Behavioral Science, University of Colorado Boulder, Boulder, CO USA; 3 University of Colorado Boulder, Department of Geography, Boulder, CO USA; 4 European Commission, Joint Research Centre, Ispra, VA Italy

**Keywords:** Environmental sciences, Social sciences

## Abstract

Understanding changes in the built environment is vital for sustainable urban development and disaster preparedness. Recent years have seen the emergence of a variety of global, continent-level, and nation-wide datasets related to the current state and the evolution of the built environment, human settlements or building stocks. However, such datasets may face limitations like incomplete coverage, sparse building information, coarse resolution, and limited timeframes. This study addresses these challenges by integrating three spatial datasets to create an extensive, attribute-rich sequence of settlement layers spanning 200 years for the contiguous U.S. This integration process involves complex data processing, merging property-level real estate, parcel, and remote sensing-based building footprint data, and creating gridded multi-temporal settlement layers. This effort unveils the latest edition (Version 2) of the Historical Settlement Data Compilation for the U.S. (HISDAC-US), which includes the latest land use and structural information as of the year 2021. It enables detailed research on urban form and structure, helps assess and map the built environment’s risk to natural hazards, assists in population modeling, supports land use analysis, and aids health studies.

## Background & Summary

Data describing the evolution of human settlements is crucial for understanding environmental change and human-environment interactions. In recent years, various spatial data products have been developed that describe the built environment at national, regional and global levels, such as Google’s building footprints^1,2^, Microsoft’s high-resolution building footprint data^3^, OpenStreetMap, and Open City Model (OCM), the Global Human Settlement Layer (GHSL)^4^, or the World Settlement Footprint Evolution^5^, to mention a few examples. While these datasets capture the human footprint in unique ways, developing comprehensive long-term settlement data has been challenging due to the lack of data and infrequent data updates.

Numerous studies conducted map matching, or geospatial data conflation, which refers to the processing of multiple sets of geospatial data to identify corresponding objects that, in combination are more reliable and allow to generate new, more comprehensive, and more complete geospatial information^6,7^. Various techniques have been proposed for matching different geospatial datasets. Geospatial objects matching often implies using distance and angular measures, shape metrics, and semantics. For example, Koukoletsos *et al*. (2012) proposed automated object-based matching, a multistage approach combining geometric and attribute constraints. Different types of remote sensing data (including aerial imagery) were combined to create spatial layers depicting built-up areas^8^.

Data products, developed using multiple geospatial input data to depict characteristics and dynamics of built-up areas and settlements, have found applications in various research fields, including population downscaling and estimating change^9,10^, the analysis of urban development^7,11,12^, the characterization of urbanization^13,14^, evaluating disaster response and recovery^15^, urban planning and land use science^16–18^, validation of remote-sensing based data^19^ and studying the impacts of natural hazards and extreme events^20,21^.

However, in addition to general aspects of uncertainty, each of the above datasets has limitations such as incomplete geographical coverage, limited information about semantic and temporal aspects, low spatial detail in historical records, or coarse spatial resolution^6,22–24^. In order to overcome such limitations and increase data quality and accuracy, recent efforts have been made to systematically integrate several disparate datasets^25,26^. Such data integration efforts are complex, and we will demonstrate the integration of several geospatial data sources to create high-quality multi-temporal data layers describing different components of the built environment. We utilized both real estate and parcel data, offering year-built information dating back to the 1800s, along with building footprint data, to create a refined, spatio-temporal representation of built-up areas. The dataset reflects the most current land use and built-up information as of 2021, and the underlying multi-source data integration will mitigate incompleteness issues in prior work^27–29^.

Herein, we describe (1) how different spatial layers can be used to extract relevant attributes for creating a multi-temporal built-up land dataset, (2) what data integration steps have to be done in a geospatial processing environment to overlay those disparate spatial datasets, (3) how to efficiently create gridded data using the integrated vector datasets as input, and (4) the creation of uncertainty surfaces and quality metrics that are relevant for future data use. The spatial resolution, reference, and extent of the resulting gridded surfaces are coherent with and represent an improved version of the existing Historical Settlement Data Compilation for the United States (HISDAC-US)^27,29^ to maintain data consistency and facilitate data use. We evaluate the data products against various independent data sources. HISDAC-US is part of a larger data integration and harmonization effort aiming to create accessible, historical settlement data at the country-level, based on (open) cadastral and other data, applied to a growing number of countries^19,30,31^.

## Methods

**Fig. 1:**
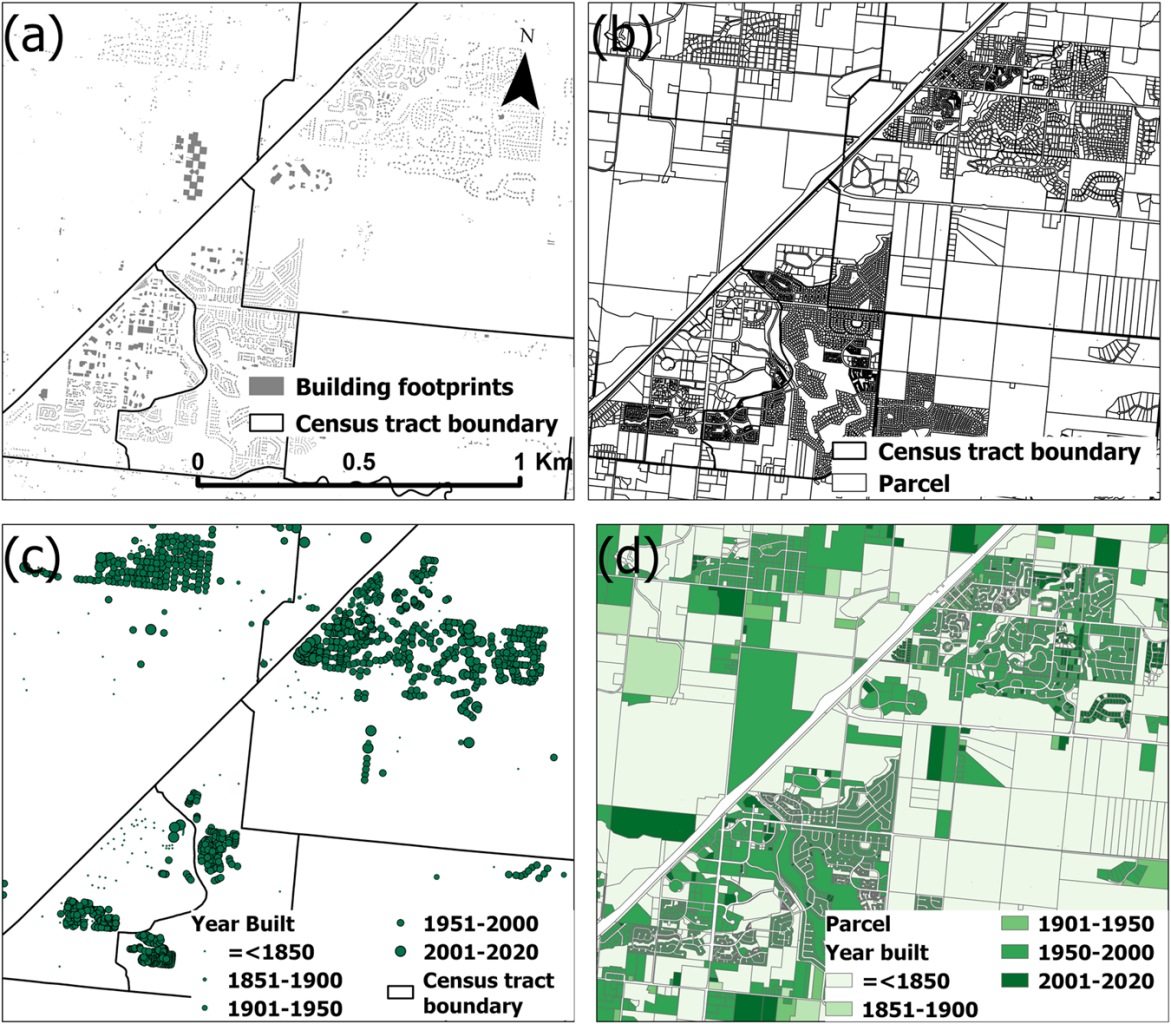
Input data illustration. (**a**) OCM building footprint data (**b**) Parcel data (**c**) ZTRAX property locations and year-built information (**d**) parcel data with year-built information.

This study integrated several datasets, including the Open City Model based on Microsoft building footprint data, nationwide parcel data from Parcel Atlas, and property data from Zillow (Zillow’s Transaction and Assessment Database; ZTRAX)^32^. Below, we describe each dataset, available attributes, and geographical coverage. Subsequently, we present the data integration process to create multi-temporal gridded surfaces/data characterizing the built environment quantitatively and by land use category (Fig. 1).

### Open city model (OCM)

Open City Model^33^ (https://github.com/opencitymodel/opencitymodel) was developed based on open datasets such as Microsoft’s building footprint data and OpenStreetMap (OSM) (Fig. 1a & Table 1) using an algorithm to estimate building heights. OCM data contains 125 million planar polygon objects representing buildings with building height information. Building heights were estimated using linear regression models based on a large sample of data for which building footprint areas and heights were available (approximately four million buildings in OSM for the U.S.).

**Table 1:** Descriptions and characteristics of input, and output.

Name	Description	Spatial resolution	Temporal resolution and time period	Data format
Building footprint data from Open City Model (OCM)	Nationwide building footprint data	—	unitemporal	polygons
Parcel Atlas	Nationwide parcel data	—	annual	polygons
ZTRAX	Nationwide property data	—	annual	points
Built-up intensity (BUI)	Cumulative gridded indoor building area in a grid cell	250 m	Semi-decadal, 1810–2020	GeoTIFF
Built-up property records (BUPR)	Cumulative gridded count of built-up property records in a grid cell	250 m	Semi-decadal, 1810–2020	GeoTIFF
Built-up property locations (BUPL)	Cumulative gridded building location counts in a grid cell	250 m	Semi-decadal, 1810–2020	GeoTIFF
Built-up area (BUA)	Cumulative gridded built-up (value 1) and not built-up (value 0) areas	250 m	Semi-decadal, 1810–2020	GeoTIFF
First Built-up Year (FBUY)	Earliest built-up year in a grid cell	250 m	Annual	GeoTIFF
Land use majority	Cumulative gridded surfaces depicting the majority land use class per grid cell	250 m	Semi-decadal, 1940–2020	GeoTIFF
Land use count	Cumulative number of buildings per land use class in a grid cell	250 m	Semi-decadal 1940–2020	GeoTIFF
Uncertainty layer	No year-built	250 m	1940–2020	GeoTIFF

### Cadastral parcel data

ParcelAtlas Features©^34^ (https://boundarysolutions.com) (Fig. 1b) contains parcel boundaries of 2,494 U.S. counties (about 77% of counties in the CONUS), encompassing 146 million current parcels provided as polygon objects (Table 1). This parcel dataset contains attributes such as unique parcel IDs, addresses, land use codes, indoor floor area, parcel area, and year-built (Fig. 1d). It also provides 16 land use categories and 340 subcategories, including agriculture, communication, commercial office, commercial retail, exempt & institutional, governmental, historical & cultural, industrial-heavy, industrial, miscellaneous, personal property, recreational, residential income - multi-family, residential, transportation, and vacant land.

### Zillow’s transaction and assessment database (ZTRAX)

ZTRAX is a large real estate database (https://www.zillow.com/research/ztrax/) (Fig. 1c & Table 1), containing 400 million detailed public records across 3,137 (about 96%) counties in the US. ZTRAX includes property characteristics (year-built, indoor area, land use), geographic information (geolocation, property address), as well as current and prior valuations, and has recently gained attention by researchers from a broad range of disciplines^35^. ZTRAX provides 12 general land use categories (agriculture, commercial, exempt, government, historical, industrial, miscellaneous, private, residential, recreational, transportation, and vacant) and 307 subcategories.

For several land use classes, low representation and lower levels of completeness were found. Therefore, five of the above classes (exempt, historical, miscellaneous, private, and transportation) were omitted from the data. Additionally, we subdivided the residential class into residential-owned and residential-income. Although some classes were omitted from the main product, they were included in creating the complementary uncertainty layers as described below.

### Data integration

**Fig. 2:**
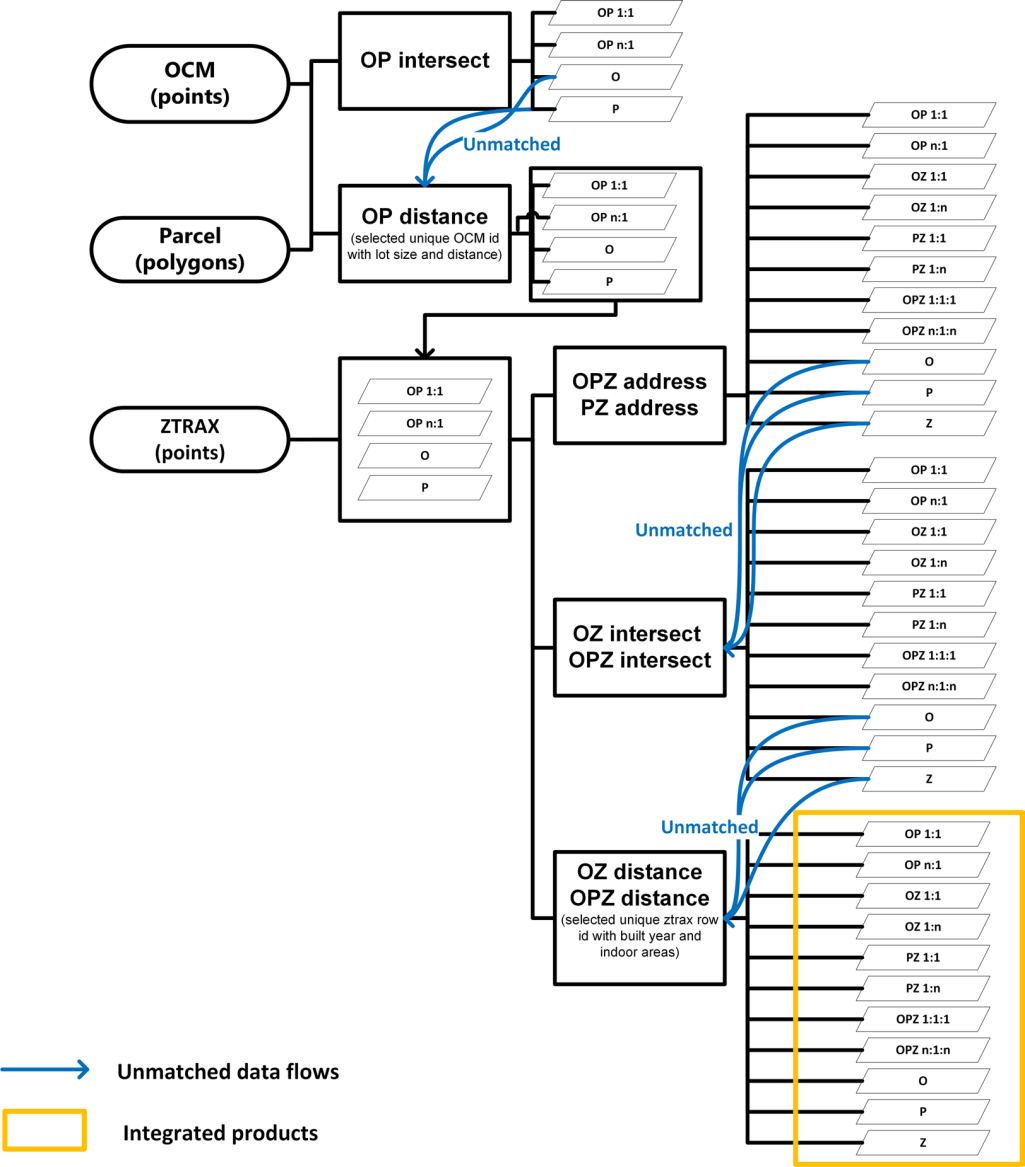
Data integration workflow (note: O: OCM, P: Parcel, Z: ZTRAX, OP: OCM building polygons merged with Parcel polygons, PZ: parcel polygons matched with ZTRAX points, OZ: OCM building polygons merged with ZTRAX, OPZ: matched OCM, Parcel, and ZTRAX).

Integrating the described spatial input data is challenging because the data is heterogeneous in its basic properties and quality parameters. The conducted processing steps aim to create a combined, higher-quality data product using the different (complementary) characteristics of the input data sources. In this case, data integration is based on various spatial operations, such as spatial join (intersection, nearest join) and address matching, to create a dataset of maximum completeness featuring attributes such as the number of buildings, building indoor area, or year-built. Due to the different data properties, the order of execution matters as described below (Fig. 2).

First, we converted OCM building footprint polygons to points (centroids) and overlaid them with parcel polygons. OCM points that were located within a parcel polygon were joined to the respective parcel record with the spatial join function in ‘geopandas’ package in Python 3.7^36^. We allowed multiple points (OCM) to join a parcel polygon (parcel), since a parcel may contain multiple buildings. Points that were not joined with a parcel based on the containment rule (because the point was located outside parcel boundaries) were joined with nearby parcel polygons (within a radius of 100 meters) if the building footprint area in the OCM record was less than the respective parcel area. If more than one point was close enough to the same polygon or more than one polygon was located within 100 m to the same OCM point, only those points with the most similar test metric were matched to enforce a 1:1 join. The test metric was calculated as shown in Eq. 1. We formulated this rule under the assumption that when a building polygon matches a parcel polygon, the building area is expected to be smaller than the parcel’s area, and building location errors should remain within the bounds of the diagonal length of the parcel polygon.

1 $${\rm{Similarity}}={\rm{Minimum}}\;\left({\rm{Normalize}}\left(\begin{array}{l}\left(\frac{Building\;footprint\;are{a}_{OCM}}{Building\;footprint\;are{a}_{parcel}}\right)\\ +\left(\frac{Minimum\;distance\;between\;OCM\;and\;Parcel}{Diagonal\;length\;of\;polygo{n}_{parcel}}\right)\\ +\left(Minimum\;distance\;between\;OCM\;and\;Parcel\right)\end{array}\right)\right)$$

Those OCM points and parcel polygons that were not matched with any record in the other dataset at that point remained unmatched. These steps resulted in the joined OCM-parcel (OP) records, composed of the successfully matched point-polygon objects (point attributes were joined to the polygon attributes), OCM points that did not match with parcel polygons (O), and parcel polygons that were not matched with points in OCM (P). We converted the O subset back to polygons to ensure we create a combined dataset with polygon geometry (Fig. 1).

Next, we integrated the OCM-parcel combined dataset (OP, O, P) with ZTRAX data. We first used address information from ZTRAX to match with records in the OP and P subsets containing addresses. The remaining ZTRAX points that were not matched yet with polygons were then examined for containment within the extended polygons (buffered by 100 meters) with the spatial join function in ‘geopandas’ package in Python^36^. If they were inside these buffered boundaries, the attributes were joined. We allowed multiple ZTRAX points to join OP, O, and P polygons. To avoid duplicate matching between a point and multiple polygons, we utilize attributes. If there are multiple polygons with attributes (year-built and indoor areas) similar to those of a ZTRAX point, we prioritize the most similar match among all the matches (Eq. 2). Those ZTRAX points that were not matched remained unmatched for the time being.

2 $${\rm{S}}{\rm{i}}{\rm{m}}{\rm{i}}{\rm{l}}{\rm{a}}{\rm{r}}{\rm{i}}{\rm{t}}{\rm{y}}={\rm{M}}{\rm{i}}{\rm{n}}{\rm{i}}{\rm{m}}{\rm{u}}{\rm{m}}({\rm{A}}{\rm{b}}{\rm{s}}{\rm{o}}{\rm{l}}{\rm{u}}{\rm{t}}{\rm{e}}(Year\,{built}_{ztrax}-Year\,{built}_{parcel})+({\rm{A}}{\rm{b}}{\rm{s}}{\rm{o}}{\rm{l}}{\rm{u}}{\rm{t}}{\rm{e}}(Indoor\,{areas}_{ztrax}-indoor\,{areas}_{parcel}))$$

Once these geoprocessing steps were executed, we integrated all the matched and unmatched records. The unmatched polygons and the Parcel-OCM polygon matches were converted to points by using polygon centroids. Matched Parcel-OCM polygons and ZTRAX points used the geometry information from Zillow. This created eleven combinations of subsets (Fig. 1) as listed below. The resulting integrated dataset consisted of 287,993,130 points. The attribute coverage varied slightly depending on the matched source datasets to generate a record. For example, we increased the completeness of data on year-built and indoor area by filling in records in ZTRAX by using those found in the parcel data. The counts for each subset created during the data integration process is detailed in Table 2. The resulting eleven subsets are:

1. Individual OCM building locations matched with individual parcel records (OP 1:1),
2. Multiple OCM building locations matched with one parcel (OP n:1),
3. Individual OCM building locations matched with individual ZTRAX records (OZ 1:1),
4. Individual OCM building locations matched with multiple ZTRAX records (OZ 1:n),
5. Individual parcels matched with individual ZTRAX records (PZ 1:1),
6. Individual parcels matched with multiple ZTRAX records (PZ 1:n)
7. Individual OCM building locations, matched with individual parcels, and individual ZTRAX records (OPZ 1:1:1),
8. Multiple OCM or ZTRAX records matched with individual parcels (OPZ n:1:n),
9. OCM building records that did not match with any other data (O),
10. Parcel records that did not match with any other data (P),
11. ZTRAX records that did not match with other data (Z).

### Creating multi-temporal gridded data

The points described above were input to the rasterization. We created a set of multi-temporal gridded surfaces/data layers using the year-built attribute for temporal definition and building locations and properties within grid cell extents to calculate and assign the cell values. To generate gridded surfaces in GeoTiff format, we used Numpy^37^ and Rasterio^38^ packages in Python. This grid is consistent with the grid used in the previous version of HISDAC-US^27^. All the gridded data layers listed in Table 1 have a spatial resolution of 250 m.

**Table 2:** Count for each integration step.

Integration code	Description	Count and percentage
O	OCM building footprint only	18,173,981 (6%)
OP11	Single OCM and parcel data match	63,963,244 (22%)
OPZ111	Single OCM, parcel and ZTRAX match	8,184,192 (3%)
OPZn1n	Multiple OCM and ZTRAX and a single parcel match	50,523,970 (18%)
OPn1	Multiple OCM building footprint and parcel match	39,296,004 (14%)
OZ11	Single OCM and ZTRAX match	15,095,266 (5%)
P	Parcel only	72,738,753 (25%)
PZ11	Parcel and ZTRAX 1:1 match	3,645,056 (1%)
PZ1n	Parcel and multiple ZTRAX match	10,155,415 (4%)
Z	ZTRAX only	3,211,091 (1%)
OZ1n	OCM and multiple ZTRAX match	3,006,158 (1%)

### Built-up area, intensity, location, and year-built data (1810–2020)

The gridded surfaces have a temporal resolution of 5 years and cover the time period from 1810 to 2020. To create the different thematic raster layers, we used the building-related attributes included in the various source datasets as described in this section. Importantly, to create the gridded layers for a given point in time, we included all the objects with a year-built equal to or prior to the point in time of interest and their attributes to compute the summary statistics and assign the grid cell values. These data products build upon previous efforts, the HISDAC-US^27,29^. Therefore, we keep the layer names and definitions consistent.

**Fig. 3:**
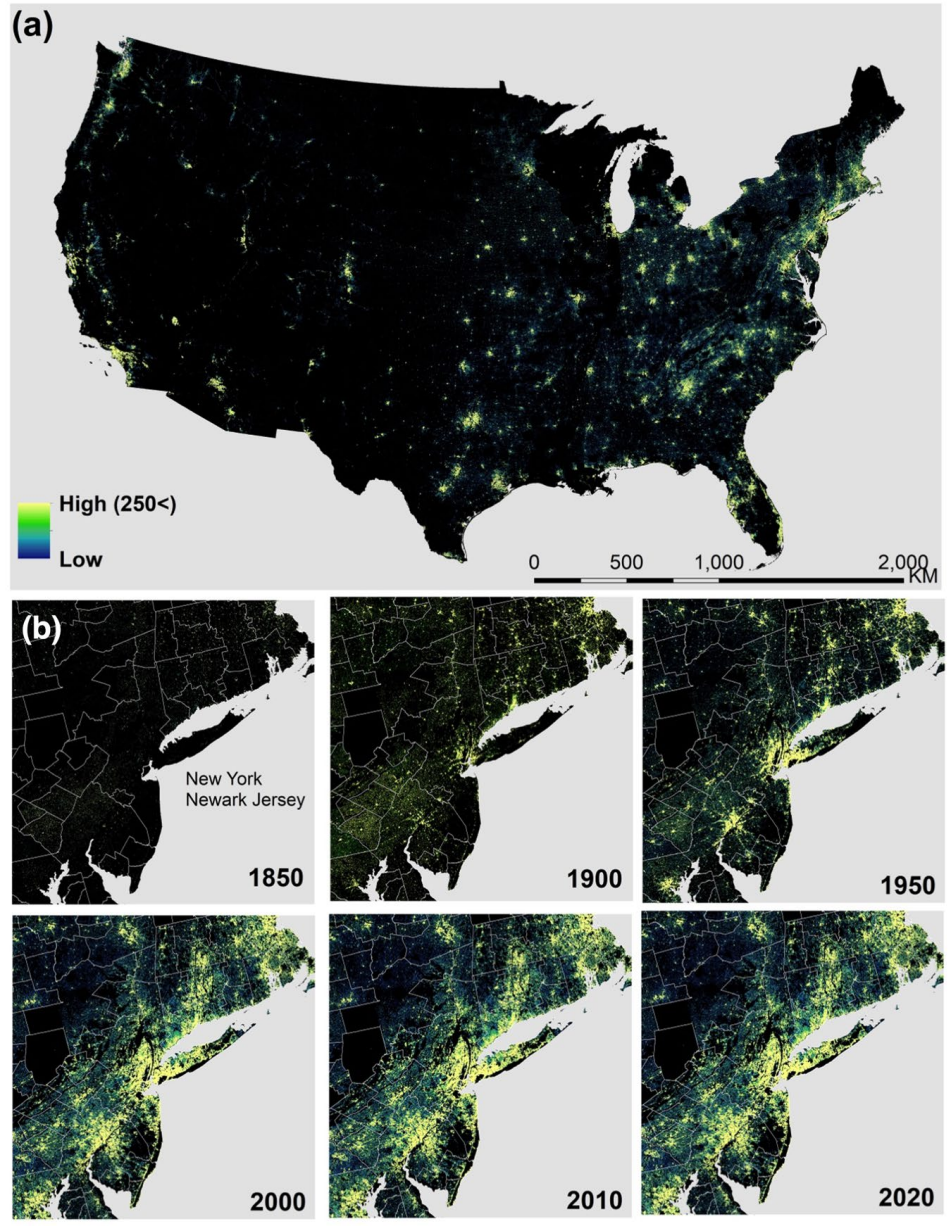
Fine-resolution time sequences of gridded building data for the US (**a**) Contemporary (2020) built-up property records (BUPR) in the US; (**b**) BUPR time sequence for the New York-Newark-Jersey area.

**Built-up area (BUA)**^29^ represents a binary representation of built-up areas. A cell that has at least one building is assigned the value ‘1’; all other areas are assigned the value ‘0’. **Built-up intensity (BUI)**^27^ is created based on the registered indoor area of all structures found within a grid cell for each half-decade (and prior to that year). Thus, BUI represents the gross (indoor) building area or the sum of the indoor areas of all units in a multi-storey building existing at that point in time. **Built-up property records (BUPR)**^29^ represents the count of property records within a raster cell at a given point in time. BUPR will allow the differentiation between areas where high-rise and multi-unit buildings dominate the built environment and other developed land. **Built-up property locations (BUPL)**^29^ represent the count of buildings per grid cell at a given point in time. BUPL is similar to BUPR in most cases. However, BUPL counts multi-family housing or apartment buildings as one built-up entity that may contain multiple units each of which is counted individually in BUPR. The **First Built-up year (FBUY)**^27^ raster layer was calculated by assigning each grid cell the earliest year-built recorded among all records within a grid cell. We used Numpy, Rasterio^37,38^, and other Python packages to generate semi-decadal gridded surfaces from 1810 to 2020 in GeoTiff format (Fig. 3).

### Land use data (1940–2020)

Due to the lower attribute completeness in records with earlier year-built but also due to inherent survival bias (see the spatial and temporal uncertainty section for more details), we created semi-decadal gridded land use data from 1940 to 2020. The HISDAC-US dataset series includes layers that represent the majority land use class within a grid cell as well as count layers for each land use category (Industrial, commercial, residential income, residential owned, agricultural, recreational, government, and vacant lands) consistent with the original HISDAC-US version^28^, all at a spatial resolution of 250 m. This data product introduced two additional land use categories, government, and vacant lands, which are considered crucial factors for analyzing urban and population dynamics as well as land development^39,40^.

To complement the original HISDAC-US land use layers, we added public and vacant land use categories. The land use categories in ZTRAX and the parcel data were matched using the Python library ‘fuzzy-match.’ Parcel and ZTRAX data exhibited closely aligned land use categorizations, with the exception of ‘Spacecraft’ in the parcel data, which did not have a corresponding match in ZTRAX. Since this land use category was unrelated to our data products, we chose to leave it unmatched.

**The agricultural** class has 25 subcategories. We included nine of these subcategories including agricultural general, farm (irrigated or dry), dairy farm, poultry farm, ranch, reservoir, water supply, rural improved, nonresidential-owned, natural resources, and miscellaneous structures. We excluded 16 subcategories that are not tied to buildings, such as range land, grazing land, cropland, field crops, row crops, orchard (fruit, nut).

**The commercial** (C) land use class includes 65 subcategories, such as office and medical buildings, dry cleaners, casinos, and gas stations. **Industrial** (I) land use contains 47 subcategories, including heavy industrial buildings such as labor camps, quarries, and slaughterhouses, and lighter industrial facilities such as loft buildings, assembly plants, and recycling centers.

**Fig. 4:**
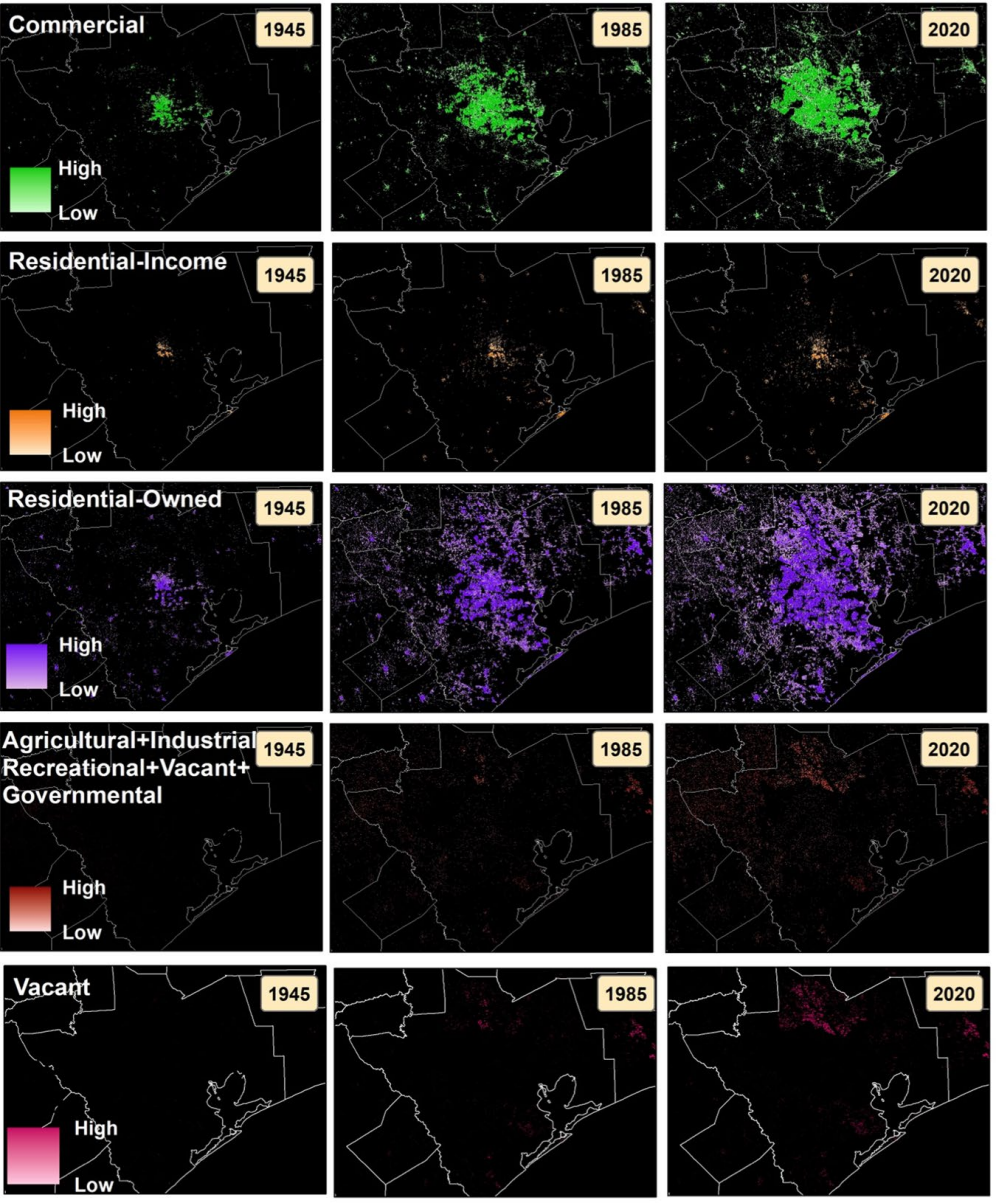
Fine-resolution time sequences of gridded landuse class counts for Austin, Texas overlaid with county boundaries (1945,1985, and 2020).

The Residential class is divided into two land use categories, residential-income and residential-own. **The residential owned** (RO) category refers to residential structures that are owned by a residential account holder who owns the property at the service address of record (https://www.lawinsider.com/dictionary/residential-owner). **Residential-income** (RI) indicates residential structures that are registered as rented or leased dwellings and, thus, not occupied by the owner. RI and RO classes combined contain 37 subcategories, all of which were included in the analysis. The **recreational land use** (RC) includes 35 subcategories, such as bowling alleys, playgrounds, zoos, and dance halls. The **government** (G) class contains 20 subcategories: city, municipal, town, village-owned buildings, administrative office, public hospitals, military, public colleges and universities, cultural, historical (monuments, homes, museums, other), and community centers. **Vacant** (V) land has 15 subcategories, including vacant land (General), abandoned site, contaminated site, under construction, structures on leased land, temporary structures, and vacant land for multi-family, agricultural, institutional and industrial use (Fig. 4).

We subset the integrated data by land use categories and year and count the occurrence of each land use class within each grid cell to create semi-decadal **land use count layers**. In addition, we created land use **majority layers** by determining the most frequently occurring land use type in each grid cell for each point in time. If the count is the same in multiple categories, we prioritized according to the order of agricultural, commercial, industrial, recreational, residential income, and residential-owned. We followed the rule which was applied for HISDAC-US (V1) to maintain consistency across data products.

### Spatial and temporal uncertainty

We created multiple uncertainty layers similar to those we created as part of the previous HISDAC-US data product. We created gridded layers representing the building structure records without year- built. A previous study investigated the potential sources of uncertainty, including incompleteness in land use attributes from earlier years in ZTRAX and the presence of survival bias, which occurs when built structures are demolished without being recorded in the data. In other words, this data relies on the assumption that the ZTRAX and Parcel data reflect the most current land use information for 2021. This information may not encompass all units that were demolished or may not fully account for historical land use changes or built-up characteristics. For instance, ZTRAX, OCM, and Parcel data do not include structures that have been demolished and do not indicate where demolitions might have taken place. Thus, omitting buildings that no longer exist at historical time points. Previous research indicates that the influence of survival bias on analytical results is limited. A recent case study showed that only about 1% of buildings were demolished in Colorado in 2015^28,32^. Nevertheless, McShane *et al*. (2022) noted that certain demolished buildings are classified as vacant. Importantly, our data product includes vacant land use, which provides valuable insights into the nature and extent of demolished buildings^28^. Furthermore, through the integration of parcel and building footprint data with ZTRAX data, the completeness of data attributes in HISDAC-US (V2) likely matches or surpasses that of HISDAC-US (V1).

## Data Records

### Historical settlement layers

The datasets described in the forthcoming sections are now accessible through the Harvard Dataverse HISDAC-US repository, which can be accessed via the following URL: https://dataverse.harvard.edu/dataverse/hisdacus^41–44^. These multi-temporal built-up characteristics are structured as georeferenced gridded layers with file names indicating the respective year and the type of attribute, including BUI, BUPL, BUPR, and BUA (e.g., 1980_BUI indicating the built-up intensity of buildings in that grid cell that existed in 1980 or before). These layers cover most of the CONUS, excluding Hawaii, Alaska, and missing counties. These datasets have a spatial resolution of 250 meters and a temporal resolution of 5 years for each data product. These datasets cover the period from 1810 to 2020.

Additionally, we have created a georeferenced gridded layer that represents the earliest construction year of buildings within a given grid cell (First Built-up Year, FBUY). To construct this layer, we gathered the earliest available year of construction data from both ZTRAX and parcel records for each property within a given grid cell. We also generated a layer that quantifies the number of buildings in a given grid cell that lacks information regarding their year of construction. This layer is denoted by the file name “NobuiltYear.”

We have provided these raster layers in the GeoTIFF format, with a consistent spatial resolution of 250 meters. To maintain uniformity across the settlement data products in the HISDAC-US compilation, we have adjusted the alignment of these layers to match the existing ones. All of this data has been made accessible through the HISDAC-US repository, utilizing the Albers Equal Area Conic projection specifically designed for the contiguous United States (EPSG:5070).

Figure 3 offers a valuable glimpse into the settlement data package, presenting distinct insights into urban development. This dataset enables users to understand the progression of urban growth by providing built-up area, intensity, and count surfaces that portray the evolution of built-up areas over time. In Fig. 3a, we present cumulative records of built-up properties across the United States. Figure 3b, on the other hand, displays a more detailed sequence of built-up property records over time in the New York-Newark-Jersey area.

### Historical land use layers

The datasets covered in the forthcoming sections have also been made accessible through the Harvard Dataverse HISDAC-US repository via the following URL: https://dataverse.harvard.edu/dataverse/hisdacus^45,46^. The multi-temporal land use majority class surfaces are structured as a series of georeferenced gridded layers, with file names incorporating the corresponding year (e.g., Majority_1940) covering most of the CONUS. These gridded layers have a spatial resolution of 250 meters and a temporal resolution of five years.

In the primary data product, each grid cell value represents the most frequently observed land use class among all ZTRAX and Parcel records within that specific grid cell for a given year. This calculation is based on all georeferenced records that include both a year-built and a land use designation. Furthermore, for each distinct land use class, we have generated a time series of gridded count layers that show the number of records associated with that land use class (e.g., residential) within a particular grid cell for every half-decade starting from 1940. These layers are labeled with the land use class and the year in their filenames (e.g., Count_1940_Theme1, indicating agricultural structures existing in 1940). These data products encompass the time span from 1940 to 2020. We have created these raster layers in GeoTIFF format, maintaining a spatial resolution of 250 meters. To ensure consistency among settlement data products within HISDAC-US, we have aligned these layers with the existing ones. All of this data has been made available in the HISDAC-US repository, utilizing the Albers Equal Area Conic projection for the contiguous United States (EPSG:5070).

Figure 4 provides an overview of the land use data package, offering unique perspectives on the evolution of land use change over time. This dataset enables users to comprehend urban growth not only in terms of the predominant themes but also through count surfaces that depict the evolution of land use classes over time. In Fig. 4, the top three rows illustrate the cumulative counts for commercial, residential-income, and residential-owned land use classes at three different time points in Austin, Texas. Meanwhile, the bottom row in Fig. 4 showcases the cumulative counts for all other land use classes, including Agriculture, Industrial, Recreational, Vacant, and Governmental.

## Technical Validation

### Attribute completeness

**Fig. 5:**
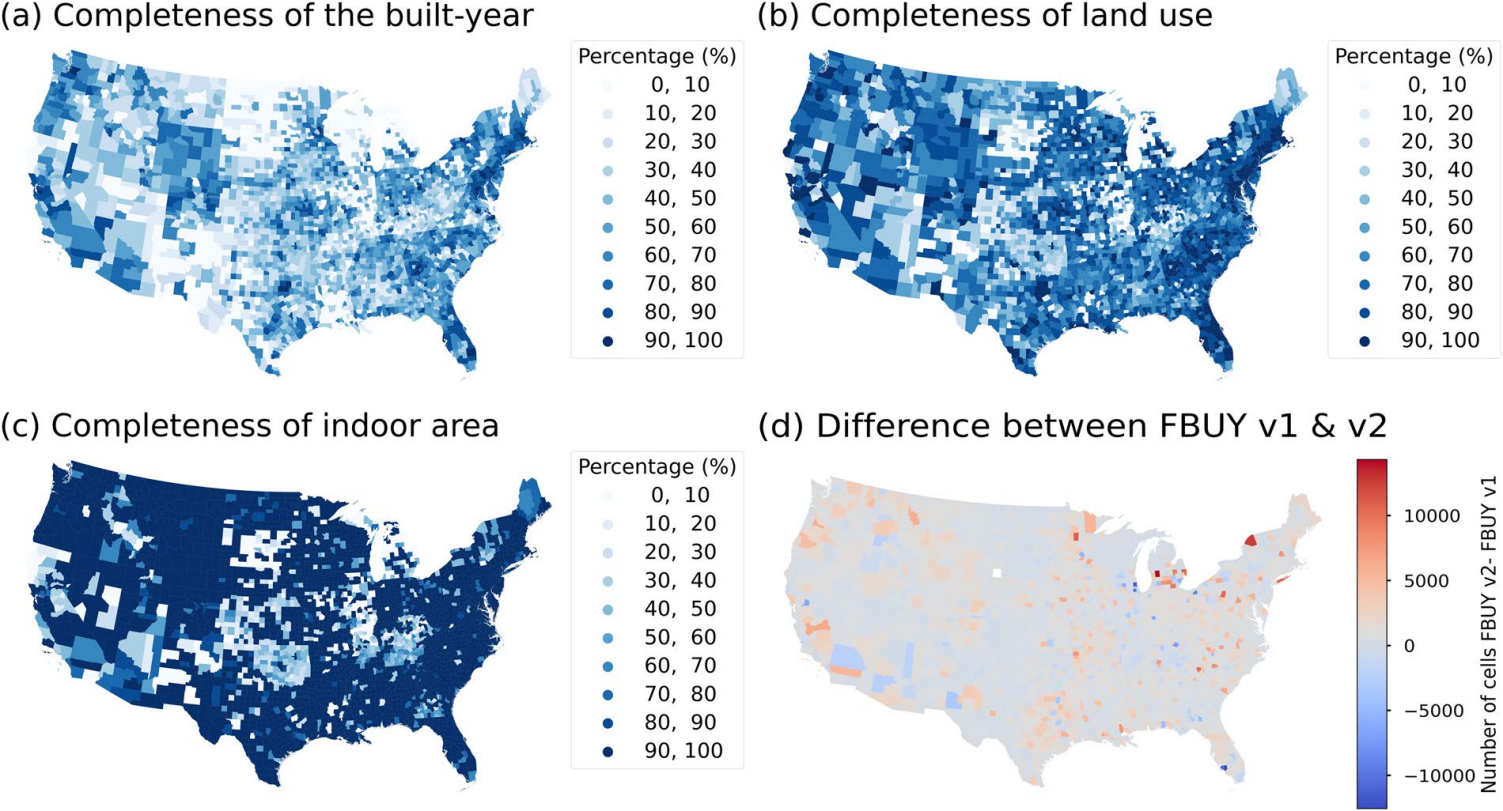
Uncertainty layers for different attributes: (**a**) Completeness of the year-built attribute at the county level (records with year-built information/ total records in each census tract), (**b**) completeness of land use attributes (records with land use information /total records in each county),(**c**) completeness of indoor area (**d**) difference between county-level FBUY derived from HISDAC-US (V2) and HISDAC-US (V1) (number of grid cells with year-built information at the county level in HISDAC-US (V2)- the same summary statistic derived from HISDAC-US (V1)).

Integrating and filling in missing information among different input data helps improve the completeness of the combined dataset. However, even the integrated data has geographically uneven levels of completeness, and this should be carefully considered as a possible ingredient for gridded layers as complimentary data products. For example, Fig. 5a illustrates the completeness of the year-built attribute and shows that some regions, such as Wyoming, New Mexico, Montana, and Louisiana, have lower levels of completeness of year-built. Figure 5b illustrates the completeness of land use data.

### Comparing the completeness in year-built information between HISDAC-US (V1) and HISDAC-US (V2)

We compared the completeness of land use and year-built information between this new version of HISDAC-US (HISDAC-US V2) and the original version of HISDAC-US (HISDAC-US V1) to better understand the improvement in coverage and completeness of HISDACUS (V2). To evaluate changes in coverage, we created binary layers from FBUY (resulting in multi-temporal BUA layers) of both HISDAC-US versions and subtracted the value of BUA of HISDAC-US (V1) from BUA of HISDAC-US (V2). In order to build BUA binary layers, cells with year-built of interest or earlier are assigned the value 1; all other cells are assigned the value 0. We then counted the number of cells with value 1 within each county and calculated the difference between the county-level summary statistics derived from both HISDAC-US versions. Figure 5d illustrates that in most parts of the conterminous U.S., year-built coverage in the integrated data product has improved. HISDAC-US (V2) exhibits an average improvement of 128 cells with year-built information across all counties, with a maximum county-level increase of 14,391 (Kent County, Michigan) cells compared to the earlier version (Fig. 5d).

### Associations between HISDAC-US versions

**Fig. 6:**
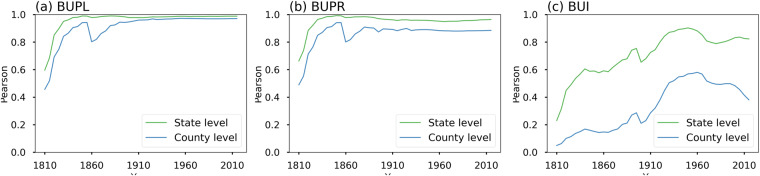
Comparison of different attributes derived from both HISDAC-US versions for the CONUS 1810–2020. Correlations for (**a**) BUPL, (**b**) BUPR and (**c**) BUI at the state and county level.

We calculated the Pearson correlations between different attributes derived from HISDAC-US (V1) and HISDAC-US (V2), including BUI, BUPL, and BUPR (Fig. 6). Correlations between BUI values vary throughout the study period and are considerably lower at the county level compared to states. BUI, BUPL, and BUPR show lower correlations in earlier years, which gradually increased over time with state-level correlations always higher than county-level correlations. The correlation trend in BUI is of particular interest as it is directly related to completeness of records in general (BUPL and BUPR) but also to attribute coverage. The weak correlations in the earlier years are due to the incompleteness of records and attribute coverage and increased significantly until the 1950s at both the county and state levels. The correlations then decrease until 2010, especially at the county level possibly due to differences in attribute coverage between both HISDAC-US versions, observations also made by Uhl *et al*. (2020) for HISDAC-US (V1).

We also compared binary FBUY layers (time slices to create BUA layers) derived from both HISDAC-US versions and conducted a confusion matrix analysis. The relative omission and commission errors showed the discrepancy between the two HISDAC-US versions. The comparison also showed that the two HISDAC-US versions have an agreement in BUA of 94% (combined true negative and true positive), a recall of 0.60, an F-score of 0.60, and a precision of 0.60.

### Evaluating historical coverage

**Fig. 7:**
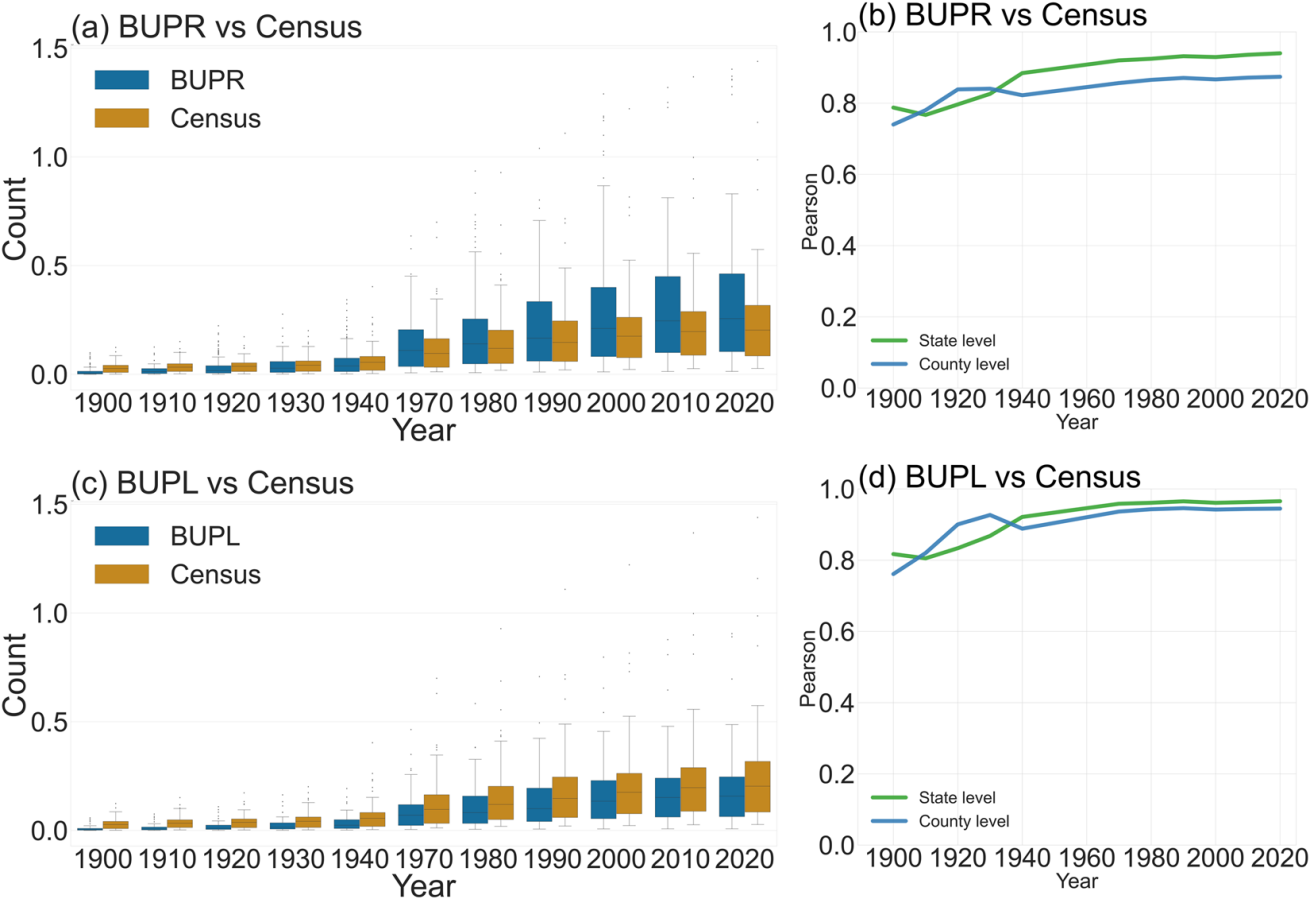
Comparison of US-wide housing trends from 1900 to 2020 based on U.S. Census data and HISDAC-US: (**a**) Comparison of state-level data distributions of BUPR and census housing counts. (**b**) Correlations between BUPR and census housing counts; (**c**) Comparison of state-level data distributions of BUPL and census housing counts. (**d**) Correlations between BUPL and census housing counts.

Additionally, we compared HISDAC-US (V2) with census data and the multi-temporal building footprint dataset (MTBF-33)^47^. MTBF-33 data covers 33 counties in the U.S. and a time period of more than 200 years up to 2015. Figure 7a,c compare data distributions of US-wide housing trends from 1900 to 2020 between the U.S. Census data and BUPR and BUPL, respectively. Figure 7b,d show the Pearson correlations over time between U.S. Census data, BUPR and BUPL, respectively, at the county and state levels. Earlier years, from 1900 to the 1940s, show similar distributions between BUPR and Census data (Fig. 7a), but the discrepancy grew in the 1970s. These results are reasonable considering BUPR data include all land use categories and census data only contains residential housing units. Moreover, Pearson’s correlation coefficient (Fig. 7b) confirms a high correlation between BUPR and the census data over time.

**Fig. 8:**
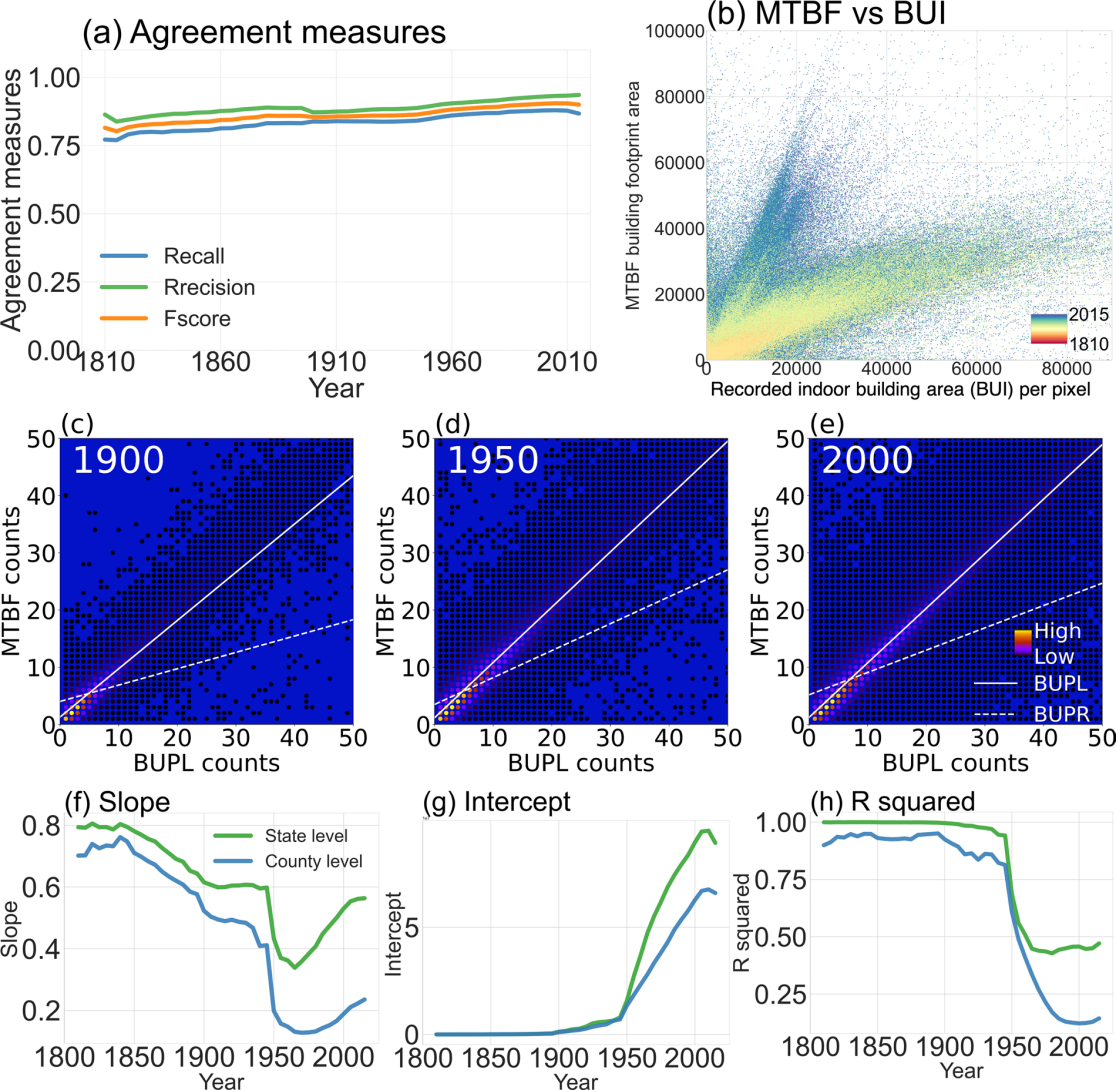
Agreement assessment between MTBF-33 and HISDAC BUPL. (**a**) Precision, recall, and F-measure between binarized MTBF-33 and BUPL over time; (**b**) the relationship between MTBF-33 building footprint area and BUI from 1810 to 2020. (**c,d**), and (**f**) illustrate the comparison of counts in MTBF-33 with counts in BUPL and BUPR for different points in time (1900, 1950 and 2000). Result of the evaluation experiments for BUI using MTBF-33 building footprint area: (**f**) Slope, (**g**) intercept, and (**h**) R^2^ of the linear regression for all points in time between 1810 and 2015 at county and state levels.

We also compared the BUI and BUPL of HISDAC-US (V2) with the building footprint area and the number of buildings over time from the MTBF-33 dataset. We conducted correlation analysis and calculated agreement measures using binarized BUPL and MTBF-33 data. Figure 8 shows the trends of Precision, Recall, and F-measure over time (Fig. 8a), indicating high levels of agreement (values between 0.7 and 0.9 for all three measures) and a significant improvement over HISDAC-US (V1)^29^. The comparison between binarized MTBF-33 and BUPL shows an overall agreement of 96% for the year 2015 and very low false positive and false negative errors that are likely induced by spatial offsets or incompleteness in the data. The MTBF-33 building footprint area and HISDAC-US (V2) BUI exhibited similar trends from 1810 to 2015 (Fig. 8b).

We conducted a comparative analysis of (non-binarized) MTBF-33 and BUPL as well as BUPR at different points in time (1900, 1950, and 2000), and found very high correlations. While in previous versions, BUPL tended to underestimate MTBF-33 when compared^29^ (Fig. 8), Fig. 8b demonstrates a robust relationship only with a minor trend of overestimations. Additionally, as expected, BUPR tends to exhibit higher counts than MTBF-33 as MTBF-33 counts represent the locations of parcels, while BUPR represents the counts of units subject to different owners, many of which can be located in one parcel. Thus, it is reasonable to observe a lower slope for BUPR.

This study also compared MTBF-33 based building footprint areas and BUI layers by running a linear regression analysis. Higher R^2^, slopes closer to one and y-intercepts closer to zero were considered better estimates. Slope, intercept, and R^2^ exhibited similar patterns to those observed for HISDAC-US (V1) as reported by Leyk and Uhl (2018). BUI in HISDAC-US (V2) demonstrated a stronger alignment with MTBF-33’s building footprint area (R^2^: 0.9 to 0.8) during the earlier years from 1810 to 1940. However, a decline in alignment was observed from 1940 onwards, which was also consistent with earlier findings. We posit that this decrease in alignment can be attributed to the increasing development of high-rise buildings since the 1940s but also the general increase of indoor area in residential housing units with similar footprint area, resulting in a growing discrepancy between the building footprints and the BUI (Fig. 8f,g,h).

### The land use proportions of HISDAC-US (v1) land use vs HISDAC-US (V2) land use

HISDAC-US (V1) land use products provide six land use classes: agriculture, commercial, industrial, residential-owned, residential-income, and recreational facilities. We converted HISDAC-US (V1) to binary data for each category in each year and compared it to the binary versions of HISDAC-US (V2).

The reader is directed to previously published works for information on further validation efforts contained in Uhl *et al*. (2020) and Mc Shane *et al*. (2022). We expand on those efforts through two additional analytical veins 1) comparing HISDAC-US (V1) to the data presented herein, and 2) assessing the accuracy of HISDAC-US (V2) using several temporal versions of the National Land Cover Dataset (NLCD)^48–50^. Given the temporal nature of the land use data and the ubiquity of NLCD usage in the academic literature, we choose to evaluate the plausibility of the HISDAC land use data at 5-years time steps against five versions of the NLCD. This assessment allows the user to better understand the nature of development within the NLCD classes and offers insight as to how to better manage elements of uncertainty contained within the land use data.

**Fig. 9:**
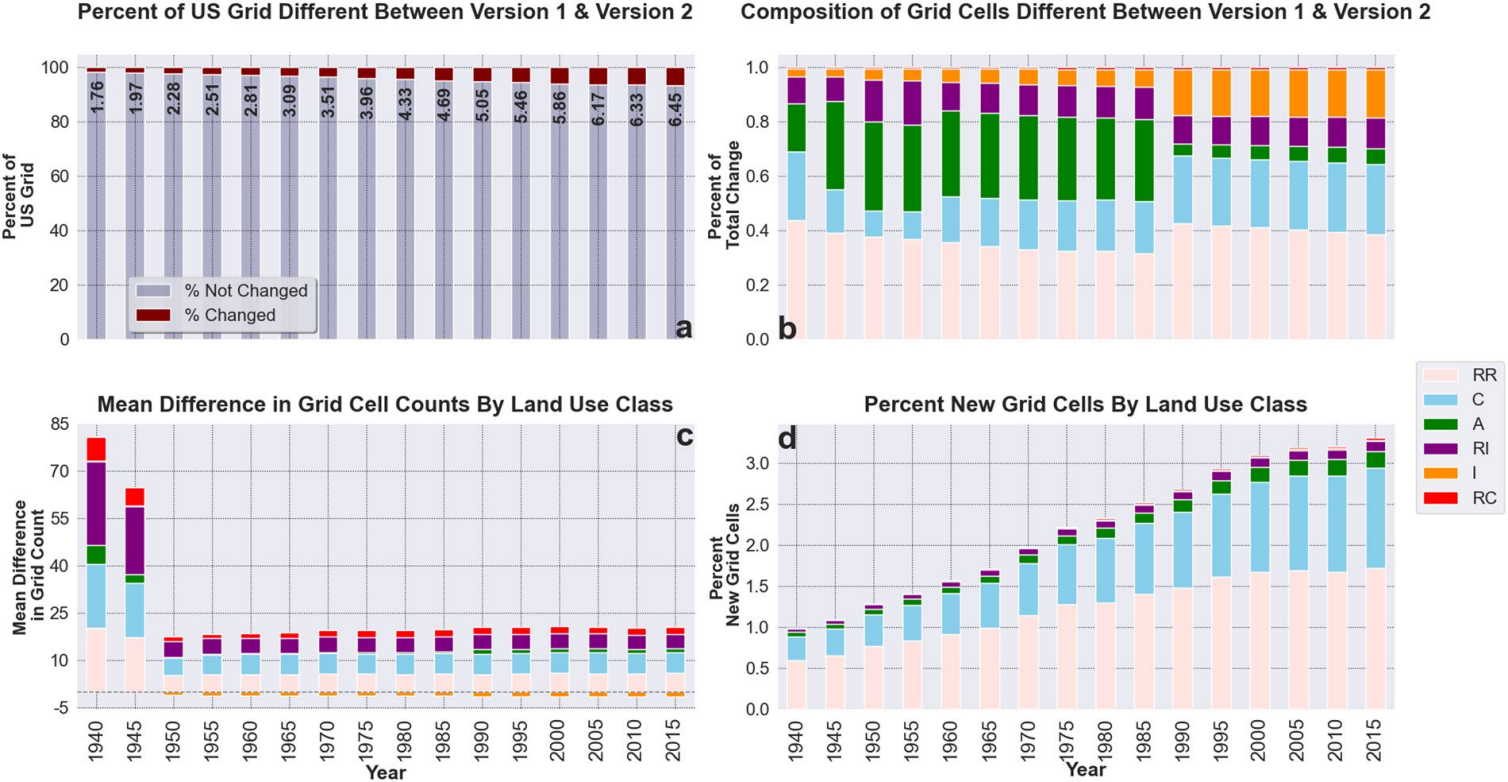
The comparison results of HISDAC-US V1 and V2 at grid cell level from 1940 to 2015 (**a**) Overall percentage of the underlying grid that was different between HISDAC-US (V1) and V2, (**b**) the land use composition of all grid cells that were different between HISDAC-US V1 and V2, (**c**) the composition of grid cells with different values and the associated value for the mean difference in grid cell counts between versions, (**d**) the percentage of grid cells that were found in HISDAC-US (V2) and not reflected in HISDAC-US (V1).

**Fig. 10:**
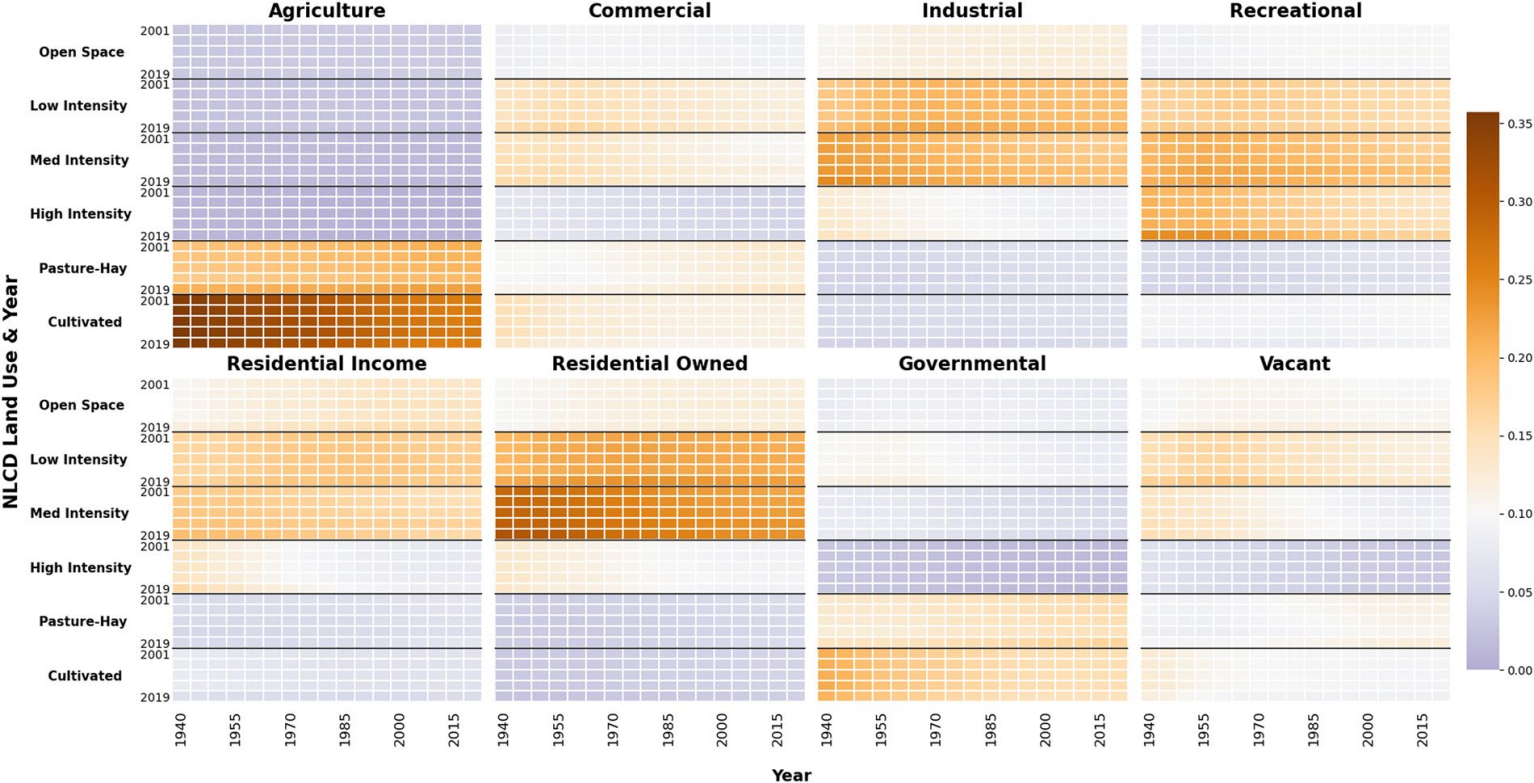
The proportion of HISDAC-US (V2) LU and NLCD LU categories from the six NLCD land cover datasets (2001, 2004, 2006, 2013, and 2019). Each panel reflects a land use category from the ZTRAX data. The Y-axis reflects each year and land use class of the NLCD data and the X-axis reflects the temporal range of the ZTRAX data. The values represent the proportion of matching records between a given NLCD land use class and ZTRAX land use class.

We find broad agreement between the two versions of HISDAC-US land use data (V1 and V2) through time. Figure 9 displays the results of the version-to-version comparison. Figure 9a quantifies the total percentage of the underlying grid that reflects different land use values in a cell-to-cell comparison. For each temporal slice (5 years) we subtracted V1 grid cell counts from V2 and calculated the percentage of grid cells (with respect to the entire grid) with a nonzero value. Figure 9b breaks down the composition of grid cell-level differences described in Fig. 9a by land use. To calculate these values, for each temporal slice and land use category, we subtracted grid values of HISDAC-US (V1) from those of HISDAC-US (V2) and calculated the percent of grid cells that held nonzero values for that land use category. Total counts and differences were then cross-checked against statistics shown in Fig. 9a to ensure accuracy. We observe relatively consistent differences in the residential-owned, residential-income, and commercial classes. Figure 9c displays the mean difference in grid cell counts by land use category. With the exception of the industrial class, all land use categories had a mean positive increase in grid cell representation. A notable trend in Fig. 9c is the significant difference between V1 and V2 between 1940 and 1950. This decade had a much larger mean increase in grid cell counts compared to other decades, although only ~1.76% of the grid changed between V1 and V2. Similarly, the total number of new grid cells in v2 for that decade represents ~1% of the overall change (Fig. 9d) between versions. To calculate Fig. 9d grid indexes were used to determine the existence of grid cells represented in V2 but not in V1. Duplicated grid indexes were subtracted from the set of IDs collected for a given temporal slice and land use category. The resulting grid IDs were then used to calculate the percentage of new grid cells (with respect to the underlying grid) represented in V2. Given the additional data and updated ZTRAX database used in V2 we assume that new grid cells augment the data observed in V1. Figure 10 displays the results for the second vein of the comparison analysis. To generate Fig. 10 five NLCD raster datasets (2001, 2004, 2006, 2013, and 2019) were reprojected and down sampled to match the 250 m grid of the HISDAC-US data sets using a majority rule aggregation function. We used a 1-hot encoding technique to then calculate the proportion of a land use category’s grid cells that aligned with a category from the NLCD grids. Six values were used from the NLCD representing developed land (21–24) and cultivated crops/pasture (81,82). An NLCD binary grid was created for each of these values. For each land use category and temporal slice in HISDAC-US (V2), a corresponding binary grid was generated indicating the presence of a land use category within a grid cell. Grid indexes for all nonzero values from the land use data were then used to extract the corresponding grid cells from the NLCD raster. We then calculated the proportion of V2 grid cells that had a nonzero value in the corresponding NLCD grid. Overall proportional representation is somewhat low, however the results are consistent with findings described in Mc Shane *et al*. (2022) and align with expected outcomes.

### Limitations

We outlined an approach for integrating various built-environment related, geospatial data sources to create a comprehensive dataset that captures built-up characteristics and land use, and their evolution over the long-term, in the conterminous United States. This involved a comparative analysis aimed at identifying biases in the built-up and land use data. However, users should be aware of several limitations when utilizing these gridded built-up data and land use datasets.

Firstly, the ZTRAX and Parcel data used herein, primarily depend on county records for land use attributes. The reporting practices among counties vary, potentially leading to omissions of existing buildings. Additionally, the land use classification methods can differ from one county to another, introducing uncertainty regarding building types. To address this, we consolidated over 300 land use types into broader thematic categories, such as commercial or residential, to reduce this uncertainty. Our dataset focuses on the land use of physical structures within identified thematic classes relevant to urban development, as per the existing literature. Consequently, the dataset does not include land uses unassociated with a structure, like cropland or grazing land, and omits other vital classifications like tax-exempt or governmental structures.

Another critical limitation is the potential exclusion of demolished buildings. The ZTRAX, OCM, and Parcel data do not account for structures that have been demolished, hence failing to represent buildings that no longer exist. For instance, cases where buildings were demolished and replaced without updating the year of construction, or where the building’s function changed post-demolition are not accurately captured. Some demolished structures might be erroneously labeled as ‘vacant.’ To mitigate this, we advise users to incorporate the vacant land gridded data in their analyses for a more comprehensive understanding.

## Usage Notes

The HISDAC-US (V2) offers a detailed gridded dataset specific to the contiguous United States, featuring a fine spatial resolution of 250 meters. This version encompasses multiple layers that detail built-up areas and land use (see table below). This dataset is versatile, suitable for various applications including urban planning, public health, climatology, and other research fields requiring high-resolution gridded data on built-up areas. The HISDAC-US (V2) dataset is available in the GeoTIFF format, ensuring compatibility with various GIS software platforms, including QGIS (http://www.qgis.org), ArcGIS (https://www.arcgis.com/), SAGA GIS (http://www.saga-gis.org/), and others. Finally, users should be aware of lower data completeness and reliability in early epochs.

**Table:** 

**File names**	**Description**
BUI.tar.gz	Archive contains the time series of BUI raster layers between 1810 and 2020 (BUI_1810.tiff … BUI_2020.tiff) with semi-decadal temporal resolution.
BUPL.tar.gz	Archive contains the time series of BUPL raster layers between 1810 and 2020 (BUPL_1810.tiff … BUPL_2020.tiff) with semi-decadal temporal resolution.
BUPR.tar.gz	Archive contains the time series of BUPR raster layers between 1810 and 2020 (BUPL_1810.tiff … BUPL_2020.tiff) with semi-decadal temporal resolution.
FBUY.tiff	The raster file (FBUY.tiff) contains the earliest year-built found in a given grid cell.
Count.tar.gz	Archive contains the time series of land use count raster layers between 1940 and 2020 with semi-decadal temporal resolution. Each land use category folder (A, C, I, RC, RI, RO, GV, and VL) contains raster layers between 1940 and 2020 (Count_1940.tiff … Count_2020.tiff).
Majority.tar.gz	Archive contains the time series of land use majority count raster layers between 1940 and 2020 (Majority_1940.tiff…Majority_2020.tiff) with semi-decadal temporal resolution.
NobuiltYear.tiff	The raster file contains the number of records without year-built.

## Data Availability

Code for analysis and validation is available at https://github.com/YoonjungAhn/HISTPLUS.
